# Dietary interventions and cognition of Alzheimer’s disease patients: a systematic review of randomized controlled trial

**DOI:** 10.1590/1980-57642020dn14-030008

**Published:** 2020

**Authors:** Sophia Camargos Moreira, Ann Kristine Jansen, Flávia Moraes Silva

**Affiliations:** 1 Universidade Federal de Minas Gerais, Ringgold Standard Institution - Belo Horizonte, MG, Brazil.; 2 Universidade Federal de Ciências da Saúde de Porto Alegre, Ringgold Standard Institution - Nutrition - Porto Alegre, RS, Brazil.

**Keywords:** Alzheimer’s disease, diet, nutrients, dietary supplements, cognition, doença de Alzheimer, dieta, nutrientes, suplementos nutricionais, cognição

## Abstract

It is estimated that by 2030 there will be 82 million people in the world with dementia.

**Objective:**

To evaluate the effect of dietary interventions on the cognitive performance of individuals with Alzheimer’s disease (AD).

**Methods:**

A systematic review of randomized controlled trials (RCT) was conducted in the Scopus, PubMed, and Cochrane databases. Thirty-two RCT were included.

**Results:**

Omega-3 fatty acid showed positive effects at different doses. Fortasyn Connect seemed to be effective in the early stages of the disease. Probiotic, Ginseng, Inositol and specialized nutritional formulas seemed to have a positive effect on cognition. Most of the primary studies presented poor methodological quality, included patients with mild AD, small samples, and did not obtain significative results for all the cognitive outcomes.

**Conclusions:**

The effect of most dietary interventions on cognition in AD patients remains inconclusive, however, several nutrients, isolated or not, show potential to improve cognitive function in AD, especially in its early stages.

## RESUMO.

Estima-se que até 2030 haverá 82 milhões de pessoas no mundo com demência.

**Objetivo:**

Avaliar o efeito de intervenções dietéticas no desempenho cognitivo de indivíduos com DA.

**Métodos:**

Foi realizada uma revisão sistemática de ensaios clínicos randomizados (ECR) nas bases de dados Scopus, PubMed e Cochrane. Trinta e dois ECRs foram incluídos.

**Resultados:**

Ácidos graxos ômega-3 apresentaram efeitos positivos em diferentes doses. O Fortasyn Connect mostrou-se efetivo em estágios iniciais. Probióticos, Ginseng, Inositol e fórmulas nutricionais especializadas também demonstraram efeito positivo sobre a cognição. A maioria dos estudos primários apresentou baixa qualidade metodológica, incluiu pacientes com DA leve e amostras pequenas e não obteve resultados significativos para todos os desfechos cognitivos.

**Conclusões:**

O efeito da maioria das intervenções dietéticas em pacientes com DA permanece inconclusivo; entretanto, vários nutrientes, isolados ou não, apresentam potencial para melhorar a função cognitiva na DA, principalmente em seu estágio inicial.

## INTRODUCTION

It is estimated that by 2030 there will be 82 million people in the world with dementia. Most of these cases occur in middle- and low-income countries that currently hold 66% of all people with the disease.^1^ Alzheimer’s disease (AD) is the leading cause of dementia, representing 50 to 70% of dementia diagnoses in the population over 65 years of age.^2^ ^,^ ^3^ In Brazil, the epidemiological data on AD are still restricted to the most developed regions of the country, where the prevalence of dementia in the elderly ranges from 7.1 to 12.9%, being AD the responsible for 55.1‒59.8% of cases.^4^ ^,^ ^5^

Categorized as a progressive neurodegenerative disorder, AD has as its main physical and anatomopathological marker the abnormal accumulation of β-amyloid peptide (Aβ) in senile plaques (SP) and hyperphosphorylated tau protein (TP) in neurofibrillary tangles (NFT), resulting in diffuse cerebral atrophy in areas of the hippocampus and frontal, parietal and temporal cortex.^2^ ^,^ ^6^ ^,^ ^7^ ^,^ ^8^ The development of the disease includes a long preclinical period, with amyloid pathology being present 15 to 20 years before the onset of cognitive decline symptoms.^2^ ^,^ ^6^ ^,^ ^9^ Risk factors for AD include advanced age, presence of Apoliprotein E4 allele genes, family history of AD and brain injury. Increasing evidence also suggests that lifestyle-related modifiable risk factors such as inadequate diet, physical and intellectual inactivity, diabetes, obesity, depression, smoking, and low education have an important role in AD due to their relationship with mechanisms involving inflammation, oxidative stress, and mitochondrial dysfunction.^3^ ^,^ ^6^ ^,^ ^7^

To date, the therapeutic resources available for AD are limited to symptom management and cannot prevent cognitive decline and disease progression. Thus, there is a growing interest in strategies that can intervene in their pathophysiological mechanisms, targeting modifiable risk factors for the disease.^6^ ^,^ ^8^ ^,^ ^10^ ^,^ ^11^ ^,^ ^12^ In the field of AD prevention, scientific evidence on the role of diet is more robust. Prospective cohort studies with the Mediterranean Diet (MeDi), Dietary Approach to Stop Hypertension (DASH), and Mediterranean-DASH Diet Intervention for Neurodegenerative Delay (MIND) in healthy subjects demonstrated a lower incidence of AD,^13^ lower rates of cognitive decline,^14^ ^,^ ^15^ ^,^ ^16^ ^,^ ^17^ cerebral atrophy^18^ and Aβ deposition^19^ in the highest adherence scores to these diets. In two other studies, both MeDi and folate and vitamin B6 consumption were inversely associated with disease incidence.^20^ ^,^ ^21^

In the field of intervention, folate, B6 vitamin and other nutrients and dietary components have been studied for their neuroprotective properties and potential positive effect on cognition. Positive results in cognitive performance have been found in experimental studies and clinical trials using omega-3 fatty acids,^8^ ^,^ ^11^ ^,^ ^12^ ^,^ ^22^ alpha-lipoic acid,^8^ ^,^ ^11^ ^,^ ^12^ polyphenols,^12^ ^,^ ^22^ ^,^ ^23^ Q10 coenzyme,^8^ ^,^ ^11^ ^,^ ^12^ vitamin supplements,^8^ ^,^ ^11^ ^,^ ^12^ ^,^ ^22^ ^,^ ^23^ selenium,^8^ and phytochemicals.^8^ ^,^ ^11^ ^,^ ^12^ ^,^ ^22^ ^,^ ^23^ However, several questions are still present regarding the reproducibility of the results found in animal models and the effectiveness of such interventions in individuals with AD in the various stages of the disease.

Therefore, considering the potential relationship between diet, cognition and AD, the present systematic review aimed to evaluate the existing evidence in controlled randomized controlled trials for the use of specific dietary interventions in the management of cognitive decline in AD patients.

## METHODS

It was a systematic review of randomized controlled trials conducted according to the protocol proposed by the Cochrane Collaboration^24^ and structured as proposed by the Preferred Reporting Items for Systematic Reviews and Meta-Analyzes (PRISMA).^25^

### Literature search strategy

The search for randomized controlled trials (RCT) was conducted in the PubMed, Cochrane Central Register of Controlled Trials, and Scopus databases, in September 2016, using a combination of the Medical Subject Heading (MeSH) terms related to the factor under study (dietary interventions in Patients with AD), outcome (cognitive performance), and design (randomized controlled trials) (Table 1). Additionally, manual searches were performed in the reference list of studies relevant to this review.

**Table 1.** Pubmed search strategy.

Descriptions of search terms used
(((((diet[Title/Abstract]) OR Dietary therapy[Title/Abstract]) OR Food habits[Title/Abstract]) OR Food formulations[Title/Abstract]) OR Food formulations[Title/Abstract]))) AND ((((Randomized Controlled Trials as Topic/) OR randomized controlled trial/) OR Random Allocation/) OR Double Blind Method/) OR Single Blind Method/) OR clinical trial/) OR clinical trial, phase i.pt) OR clinical trial, phase ii.pt) OR clinical trial, phase iii.pt) OR clinical trial, phase iv.pt) OR controlled clinical trial.pt) OR randomized controlled trial.pt) OR multicenter study.pt) OR clinical trial.pt) OR exp Clinical Trials as topic/))))) AND Alzheimer disease[Title/Abstract]

### Selection of eligible studies

The studies identified in the search in the three databases were stored in the reference organizer program Endnote Web, after deleting duplicates. Two reviewers (AKJ and SHCM) independently assessed the titles and abstracts and the discrepancies were resolved by a third reviewer (FMS). After the selection of potentially eligible articles, a reviewer (SHCM) read the full texts for confirmation of inclusion in this systematic review and collected the data from a standardized form.

The following criteria were considered eligible for the two stages of study selection:

- A randomized clinical trial design.
- Including participants with a prior diagnosis of AD.
- Having assessed cognition as primary or secondary endpoint.
- Having had interventions such as diet or food or specific supplement.

Studies that included participants with different types of dementia and did not present stratified results for AD were excluded, as were those whose presented open-label phase results from an included study and whose full text could not be accessed. Only studies whose manuscript were published in English were selected.

#### Data extraction and methodological quality assessment of eligible studies

The data extracted from the articles were:

- Authorship.
- Date of publication.
- Country where the study was conducted.
- Study design.
- Method of randomization.
- Blinding of participants, researchers, and evaluators.
- Sample size and proportion of subjects completing the study.
- Inclusion and exclusion criteria.
- Criteria used to diagnose ad.
- Mean age of participants.
- Proportion of male subjects.
- Dietary intervention protocol.
- Cognitive outcomes analyzed.
- Main results.

To evaluate the methodological quality, the criteria proposed by Cochrane were evaluated from the six domains: randomization method, allocation concealment, blinding scheme (participants, professionals, and outcome assessors), intention-to-treat analysis (ITT), follow-up losses and selection of outcomes.^24^

## RESULTS

**Figure 1.**
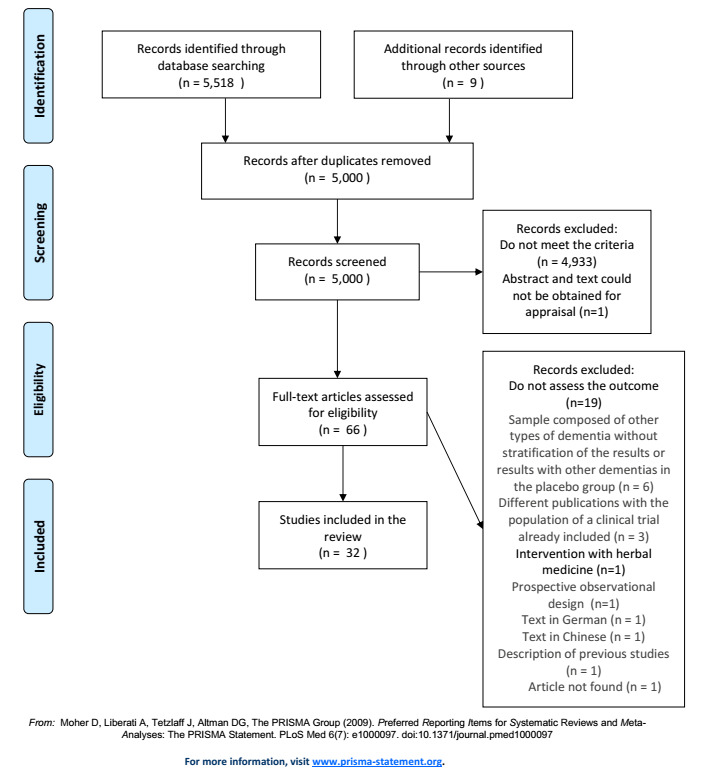
Prisma flow diagram.

From the search in the three databases, 5,000 articles were identified, after duplicate exclusion. Additionally, nine articles considered relevant to the study were included. Thirty-two studies met the eligibility criteria and were included in this review. The study flowchart is presented in Figure 1.

### General characteristics of studies

The overall characteristics of the selected studies are presented in Table 2. The majority were conducted in the United States (41.0%) and in European countries (34.0%). The sample size ranged from 12 to 613 individuals per test with a follow-up period of 25 weeks in men, ranging from three weeks to thirty months. The mean age of participants ranged from 66.1 to 84.8 years and in seven studies (22%), more than half of the sample consisted of men.

**Table 2.** Characteristics and results of randomized clinical trials evaluating dietary interventions in Alzheimer’s disease.

Study (Year)^Ref^	Delimitation Place Duration of intervention	Sample Diagnostic Criteria Mean MMSE at baseline	Interventions	Cognitive outcomes	Results and main findings
ORAL NUTRITIONAL FORMULATIONS					
Lauque et al. (2004)^26^	RCT, parallel, single-blind, controlled France 6 months	39 subjects (78.8+5.4 years old, proportion of individuals by gender not described) Diagnosis of AD by NINCDS/ADRDA criteria MMSE=15.2±8.2	IG: Usual nutritional care+hypercaloric oral nutritional supplement enriched with proteins, vitamins and minerals, offering an additional 300 to 500 kcal per day. CG: usual nutritional care. Obs.: CG patients who received oral supplementation during the study were not excluded, but prescriptions were recorded. Period of intervention: 3 months.	MMSE	There was no significant difference between groups in the change in MMSE scores after 3 and 6 months of intervention compared to the baseline.
Planas et al. (2004)^27^	RCT, parallel, double-blind, placebo-controlled Spain 6 months	44 subjects (74.6±8.1 years old, 45% men) Diagnosis of AD by NINCDS/ADRDA criteria	IG: ONS hypercaloric and hyperproteic, 2 times daily. Composition: 500 kcal, 45% carbohydrates, 25% lipids, 30% protein, additional nutrients: 38 mg of Î±-tocopherol, 250 mg of vitamin C, 1.5 ìg of B12, 200 ìg of folate, 10 mg of Zn, 1,500 ìg of Cu, 3 mg Mn, 15% WheyProtein, 3.5 g arginine. CG: placebo isocaloric, 2 times daily with the same distribution of macronutrients. Composition: 5 mg of α-tocopherol, 30 mg of vitamin C, 0.38 μg of B12, 52 μg of folate, 5 mg of Zn, 500 μg of Cu, 1.25 mg of Mn, 0% of WheyProtein, 0 g of arginine.	MEC and Set	There was no significant difference between groups in the change in MMSE scores after 3 and 6 months of intervention compared to the baseline.
Salas-Salvadó et al. (2005)^28^	RCT, parallel, opened, controlled Spain 3 months	53 subjects (84.8+6.8 years old, 17% men) Diagnosis of AD by DSM-IV criteria	IG: Complete dietary formula, semi-solid or liquid, based on frozen-dried foods (Vegenat®-med), replacing breakfast, lunch and supper+dietary guidelines. CG: dietary guidelines similar to IG.	GDS scale and Pfeiffer test	No statistically significant difference was found between the groups in the change in Mini-cog and Set scores after 6 months of treatment.
Scheltens et al. (2010)^29^	RCT, parallel, double-blind, placebo-controlled Holland, Germany, Belgium, United Kingdom and United States. 12 weeks with possible 12-week extension 24 weeks	225 subjects (73.7+7.5 years old, 50% men) Diagnosis of Probable AD by the NINCDS-ADRDA criteria MMSE=20‒26	IG: Bottle of Souvenaid®, once a day (300 mg of EPA+1,200 mg of DHA+106 mg of phospholipids+400 mg of choline+625 mg of uridine monophosphate+40 mg of vitamin E+80 mg of vitamin C+60 mcg of selenium+3 mg of vitamin B12+1 mg of vitamin B6+400 mg of folic acid) CG: isocaloric placebo.	WMS-r, ADAS-cog and MMSE	Significant difference was found between groups on the percentual of cognitive decline evaluated with WMS-r immediate recall. No statistically significant difference was observed for the remaining outcomes. Changes on WMS-r immediate recall test after 12 weeks (p=0.021) IG: Decline=19%; No change=41%; improvement=40% CG: Decline=45%; No change=15%; Improvement=40%
De Sousa et al. (2012)^30^	RCT, parallel, nonblinded Portugal 90 days	37 subjects (78.9+6.1 years old, 26% men) Diagnosis of early AD by DSM-IV and ICD-10 MMSE=17‒18	IG: ONS, providing 400 kcal, 42.8 g of carbohydrates, 17.4 g of lipids and 18 g of proteins per day+standard dietary advice. IG: standard dietary advice. Period of intervention: 21 days.	MMSE and CLOX-1	There was no significant difference between the groups in the MMSE and CLOX-1 scores. Differences in scores relative to the baseline were not computed in both tests.
Scheltens et al. (2012)^31^	RCT, parallel, double-blind, placebo-controlled Netherlands, Germany, Belgium, Spain, Italy, and France 24 weeks	259 subjects (73.8+7.7 years old, 51% men) Diagnosis of Probable AD by the NINCDS-ADRDA criteria	IG: Bottle of Souvenaid®, once a day (300 mg of EPA+1,200 mg of DHA+106 mg of phospholipids+400 mg of choline+625 mg of uridine monophosphate+40 mg of vitamin E+80 mg of vitamin C+60 mcg of selenium+3 mg of vitamin B12+1 mg of vitamin B6+400 mg of folic acid). CG: isocaloric placebo.	NTB (z-score): memory function, executive function and total score.	A significant difference was found between the groups in the memory domain of the NTB scale and a trend to effect on the total NTB composition score after 24 weeks of treatment. No difference was found on the executive function. NTB memory domain - Z-score (p=0.023) IG: baseline=-0.021+0.812 12 weeks=0.089±0.381 24 weeks=0.202±0.395 CG: baseline=0.078+0.884 12 weeks=0.143±0.429 24 weeks=0.111±0.463 NTB total compositions - Z-score (p=0.053) IG: baseline=0.029+0.695 12 weeks=0.03±0.284 24 weeks=0.120±0.278 CG: baseline=0.115+0.719 12 weeks=0.075±0.262 24 weeks=0.035±0.28
Shah et al. (2013)^32^	RCT, parallel, double-blind, placebo-controlled United States 24 weeks	528 subjects (76.7+8.2 years old, 48% men) Diagnosis of Probable AD by the NINCDS-ADRDA criteria MMSE=14‒26	IG: Bottle of Souvenaid®, once a day (300 mg of EPA+1,200 mg of DHA+106 mg of phospholipids+400 mg of choline+625 mg of uridine monophosphate+40 mg of vitamin E+80 mg of vitamin C+60 mcg of selenium+3 mg of vitamin B12+1 mg of vitamin B6+400 mg of folic acid) CG: isocaloric placebo.	ADAS-cog, CDR-SOB and cognitive test battery	No significant difference was found between the groups in the rates of change of all cognitive outcomes after 24weeks of treatment.
Soininen et al. (2017)^33^	RCT, parallel, double-blind, placebo-controlled Finland, Germany, Netherlands And Sweden 24 months	311 subjects (71.9±6.6 years old, 50% men). Diagnosis of prodromal Alzheimer disease according to the IWG-1 classification and NIA-AA criteria MMSE≥24	IG: Bottle of Souvenaid®, once a day (300 mg of EPA+1,200 mg of DHA+106 mg of phospholipids+400 mg of choline+625 mg of uridine monophosphate+40 mg of vitamin E+80 mg of vitamin C+60 mcg of selenium+3 mg of vitamin B12+1 mg of vitamin B6+400 mg of folic acid) CG: isocaloric placebo.	- NTB composite score - NTB total score, memory function and executive function - CDR-SOB - Progressiontodementia (DSM-IV and NINCDS-ADRDA - Brain volumes (MRI)	There was no significant difference between the groups in changing the NTB score in relation to the baseline, the primary outcome of the study. There was a difference for CDR-SOB, hippocampal volume and ventricular volume, the latter assessed by MRI. Changes on CDR-SOB after 24 months (p=0.005) IG: 0.56+1.32 CG: 1.12+1.72 Changes on hippocampal volume after 24 month (p=0.005) IG: -0.30+0.27 CG: -0.43+0.33 Changes on ventricular volume after 24 months (p=0.046) IG: 5.96+4.66 CG: 7.80+5.53
OMEGA-3 FATTY ACIDS ISOLATED OR IN ASSOCIATION WITH OTHER NUTRIENTS					
Yehuda et al. (1996)^34^	RCT, parallel, double-blind, placebo-controlled Israel 4 weeks	100 subjects (50 to 73 years old, 79% men) MMSE=7.8±3.8	IG: 1 mL of SR-3 formulation (0.25 mL mixture of a-linolenic and linoleic acids in ratio of 1:4.5; 0.73 mL of mineral oil; and 0.02 mL α-tocopherol), twice a day. CG: 1 mL of placebo (mineral oil+α-tocopherol), twice a day.	12-item questionnaire, completed by caregivers, including areas of spatial orientation; cooperation; humor; appetite; organization; short-term memory; long-term memory; sleep disorders; alertness during the day; hallucinations; capacity for expression; and bladder control.	For all items of the questionnaire, the percentages of improvement in the IG were higher, however the level of statistical significance of this finding was not presented. Regarding the evaluation of the effects of the intervention by the caregivers, a greater number of reports of improvement in the conditions of the patients was found in the IG (p<0.001). IG: 0 reported worsening, 11 observed no difference, and 49 reported improvement. CG: 5 reported worsening, 30 did not observe any difference, and 5 reported improvement. Obs.: blinded caregivers.
Freud-Levi et al. (2006)^35^	RCT, parallel, double-blind, placebo-controlled Sweden 12 months	204 subjects (74+9 years, 48% men) Diagnosis of AD by DSM-IV criteria.	IG: omega-3 in capsules, 4,000 mg per day (1,700 mg of DHA+600 mg of EPA), added with 16 mg of vitamin E. CG: Corn oil in capsules, 4,000 mg per day, added with 16 mg of vitamin E. 6 months of placebo-controlled intervention, followed by 6 months with omega-3 supplementation for both groups (4,000 mg per day).	MMSE, ADAS-cog, CDR and CDR-SOB.	There was no significant difference between the groups after 6 months and 12 months in the MMSE, ADAS-cog, CDR and CDR-SOB. In subgroups with mild AD (MMSE >27 points) (n=32), IG individuals showed a smaller decline in MMSE in the first 6 months compared to the control group. In the other cognitive tests, the difference was not statistically significant. Subgroup with mild AD: MMSE (p=0.02) IG: baseline=28.4, 95%CI 28.1-28.7 6 months=27.9, 95%CI 27.1-28.7 12 months=27.3, 95%CI 26.1-28.4 GC: Baseline=28.5, 95%CI 28.2-28.9 6 months=26.0, 95%CI 24.2-27.8 12 months=25.4, 95%CI 23.3-27.5 Rate of decline in MMSE at 6 months (p=0.01) IG: -0.5 points CG: -2.6 points GC decline in the MMSE in the two periods (0-6 months and 6-12months) 0-6 months= -2.6 points (p<0.001) 6-12 months= -0.83 points (p=0.23)
Quinn et al. (2010)^36^	RCT, parallel, double-blind, placebo-controlled United States 18 months	402 subjects (76.0+8.7 years old, 48% men) Prior diagnosis of probable AD.	IG: DHA derived from algae in capsules, 2,000 mg per day (45‒55% of the DHA weight). CG: placebo (corn or soybean oil).	ADAS-cog, MMSE, CDR-SOB.	No significant difference was found between the groups in the rates of change of the ADAS-cog, CDR-SOB and MMSE scores after 18 months of treatment. In the analysis of subgroups with and without the APOE ε4 allele in the APOE å4 negative group, subjects receiving DHA supplementation (n=61) had a significantly lower decline in ADAS-cog and MMSE compared to those receiving placebo (n=48), whereas in the other outcomes the differences were not statistically significant. In the APOE ε4 positive group, there was no significant difference between the groups in any outcome evaluated. Subgroup APOE ε4 negative: Changing in ADAS-cog after 18 months (p=0.03) IG: 6.23 points,95%CI 4.08‒8.38 CG: 10.11 points, 95%CI 7.12‒13.1 Changing in MMSE after 18 months (p=0.03) GI: -3.36 points, 95%CI 2.16‒4.56 GC: -5.12 points; 95%CI 3.70‒6.54
Shinto et al. (2014)^37^	RCT, parallel, double-blind, placebo-controlled United States 12 months	39 subjects (75.9+9.8 years old, 56% men). Diagnosis of AD by DSM-IV criteria.	IG-1: omega-3 in capsules, 3,000 mg per day (675 mg of DHA+975 mg of EPA)+placebo from ALA. IG-2: omega-3 in capsules, 3,000 mg per day (675 mg of DHA+975 mg of EPA)+ALA in tablets, 600 mg per day. CG: Placebo of omega-3+placebo from ALA.	MMSE and ADAS-cog	In the comparison with the CG, after 12 months of intervention only the IG-2 presented smaller cognitive decline evaluated by the MMSE. There was no statistically significant difference in the decline assessed by the ADAS-CGI in the IG-1 *vs*. CG and IG-2 *vs*. CG. MMSE IG-1: -4.3+1.3 points (p=0.80) IG-2: -1.0+0.7 points (p<0.01) CG: -4.6+1.4 points
MICRONUTRIENTS ISOLATED OR IN ASSOCIATION					
Sano et al. (1997)^38^	RCT, parallel, double-blind, placebo-controlled United States 24 months	341 subjects (73.4± 8.0 years old, 35% men) Diagnosis of Probable AD by the NINCDS-ADRDA criteria MMSE=12.6	IG-1: 10 mg of Selegiline+2,000 IU of α-tocopherol per day. IG-2: Selegiline placebo+2,000 IU of α-tocopherol per day. IG-3: Placebo of α-tocopherol+10 mg of Selegiline per day. CG: Selegiline placebo+α-tocopherol placebo.	ADAS-cog and MMSE	There was no significative difference among the groups in both outcomes.
Sun et al. (2007)^39^	RCT, parallel, double-blind, placebo-controlled Taiwan 26 weeks	89 subjects (74.8+7.3 years old, 50.6% men) Diagnosis of AD by DSM-IV MMSE=10‒26.	IG: Methylcobalamin, 0.5 mg, once a day+multivitamin supplement once a day (1 mg of folic acid, 5 mg of pyridoxine hydrochloride, 60 mg of iron carbonate, 10 mg of nicotinamide, 250 mg of calcium carbonate, 2 mg of riboflavin, 3 mg monohydrate thiamine, 1 mg calcium pantothenate, 100 µg ascorbic acid, 100 µg iodine, 150 µg copper, 3 µg B12, 4,000 IU vitamin A, 400 IU vitamin D3). CG: placebo.	ADAS-Cog/11, MMSE and CASI	There was no significative difference among the groups in all outcomes.
Kessler et al. (2008)^40^	RCT, parallel, double-blind, placebo-controlled Germany 12 months	68 subjects (69.5±7.3 years old, 44% men) Diagnosis of Probable AD by the NINCDS-ADRDA criteria MMSE=20‒25.	IG: 51.62 mg of Cu-(II)-orotate-dihydrate per day, corresponding to 8 mg Cu. CG: placebo.	ADAS-cog and MMSE	There was no significant difference between the groups in the analysis of time x treatment interaction for both ADAS-cog and MMSE.
Aisen et al. (2008)^41^	RCT, parallel, double-blind, placebo-controlled United States 18 weeks	409 subjects (76.3+8.0 years old, 44% men) Prior diagnosis of probable AD MMSE=14‒26.	IG: 5 mg of folic acid+1 mg of vitamin B12+25 mg of pyridoxine per day. CG: placebo tablet.	ADAS-cog, MMSE, CDR-SOB	There was no significant difference between groups in the rate of decline of ADAS-cog, MMSE, CDR-SOB during treatment.
Lloret et al. (2009)^42^	RCT, parallel, double-blind, placebo-controlled Spain 6 months	75 subjects (mean age and proportion of individuals by gender not described). Diagnosis of AD by the NINCDS-ADRDA criteria Individuals at different stages of the disease (mild, moderate and severe dementia)	IG: 800 IU/dia de Vitamina E. CG: placebo.	MMSE, CLOX-1 and Blessed-Dementia Scale,	There was no significant difference between IG and CG in the analyzed outcomes. In analysis of IG subgroups with respondents (RP) and non-respondents (NRP) patients*, when comparing both, NRP showed a decline in MMSE (p<0.05). When comparing subgroups with placebo, NRP also declined (p<0.05). *NRP=showed no decline in serious levels of oxidized glutathione after treatment. *RP=showed a decline of oxidized glutathione after treatment. Note: Results presented graphically. Values not reported by the authors.
Remington et al. (2009)^43^	RCT, parallel, single blind, placebo-controlled United States 12 months	12 subjects (mean age and proportion of individuals by gender not described) Diagnosis of Probable AD by the NINCDS-ADRDA criteria MMSE=11.9±2.5	IG: Nutraceutical Formulation, 2 tablets per day (400 ìg of folic acid, 6 ìg of vitamin B12, 30 IU of α-Tocopherol, 400 mg of S-Adenosyl-Methionine, 600 mg of N-Acetyl-Cysteine, 500 mg of Acetyl-L-Carnitine). CG: placebo.	DRS-2 and CLOX-1	There was no significant difference between the groups in the comparison of the total DRS-2 and CLOX-1 scores after treatment.
Galasko et al. (2012)^44^	RCT, parallel, single blind, placebo-controlled United States 16 weeks	78 subjects (72.7±9.0 years old, 54% men) Diagnosis of Probable AD by the NINCDS-ADRDA criteria MMSE≥16	IG-1: 800 IU per day of vitamin E (α-tocopherol)+500 mg per day of vitamin C+900 mg per day of ALA (E/C/ALA). IG-2: 1200 mg of CoQ per day. CG: placebo.	MMSE	A greater cognitive decline was observed in IG-1 when compared to placebo. MMSE: GI-1: -2.8+2.9 (p=0.02) GI-2: -1.0+2.5 (p>0.05) Placebo: -0.9+2.5
Dysken et al. (2014)^45^	RCT, parallel, single blind, placebo-controlled United States 2.3 years	613 subjects (78.8+7.1 years old, 97% men) Diagnosis of Possible or Probable AD by the NINCDS-ADRDA criteria MMSE=12‒26	IG-1: 2,000 IU α-Tocopherol+20 mg memantine per day. IG-2: 2,000 IU α-Tocopherol per day+memantine placebo. IG-3: 20 mg of memantine per day+α-Tocopherol placebo. IG-3: 20 mg de Memantina por dia+placebo de á-Tocoferol. CG: placebo of α-Tocopherol+placebo of Memantine.	ADAS-cog and MMSE	There were no significant differences between the groups in the MMSE and ADAS-cog scores.
Nolan et al. (2015)^46^	RCT, parallel, double-blind, placebo-controlled Ireland 6 months	62 subjects (78.0+7.2 years old, 50% men) Diagnosis of mild to moderate AD defined as MMSE score between 14 and 24 with documented difficulty in other cognitive domains	Two branches of the study: individuals with AD and individuals without AD (age-matched controls). Both received intervention or placebo. IG: Supplement Macushield® - (10 mg of meso-zeaxanthin+10 mg of lutein+2 mg of zeaxanthin per day). CG: placebo.	MMSE	There was no statistically significant difference in MMSE after 6 months of treatment in the two branches of the study (individuals with AD and individuals without AD).
Remington et al. (2015)^47^	RCT, parallel, single blind, placebo-controlled United States 9 months	141 subjects (77.8+8.4 years old, proportion of individuals by gender not described) Previous diagnosis of AD, diagnostic criterion not described MMSE=22.2+5.1	IG: Nutraceutical Formulation, 2 tablets per day (400 ìg of folic acid, 6 ìg of vitamin B12, 30 IU of α-Tocopherol, 400 mg of S-Adenosyl-Methionine, 600 mg of N-Acetyl-Cysteine, 500 mg of Acetyl-L-Carnitine). CG: placebo. Treatment period: 3 or 6 months.	CLOX-1 and DRS-AEMSS	390/5,000 After 3 months, only the IG showed a statistically significant increase in the CLOX-1 scores (p=0.0002; 95%CI 0.8727‒2.6273) and DRS-AEMSS (p<0.0001; 95%CI 1.2363‒3.2283) compared to the baseline. Results presented graphically as mean+SD of the change in scores in each group. Mean IG and CG scores at baseline and at 3 months were not reported by the authors.
GINSENG					
Lee et al. (2008)^48^	ECR, parallel, open, controlled South Korea 24 weeks	97 subjects (66.1+9.1 years old, 34% men) Diagnosis of Probable AD by the criteria of NINCDS-ADRDA MMSE=21.5‒22.0	IG: conventional treatment+4.5 g White Korean powder Ginseng a day. Obs.: in addition, 9 patients were treated with 9.0 g of Ginseng (GI-2) a day to evaluate any possible effect of dose-response. CG: only conservative and supportive treatment. Period of intervention: 12 weeks.	ADAS-cog and MMSE	In comparison with control, the groups treated with Ginseng presented improvement in the cognitive performance (ADAS-cog and MMSE) during 12 weeks of treatment, being eliminated 12 weeks after its discontinuation. There was no difference in the effect of both Ginseng dosages on the cognitive performance (comparison IG *vs*. IG-2). MMSE (change of score) - After 4 weeks of treatment (p=0.033) IG: 1.0+2.4 CG: -0.58+2.4 - After 12 weeks of treatment (p=0.009) IG: 1.8+2.8 CG: -0.03+3.1 - 12 weeks after discontinuation (p=0.673) IG: 0.56+3.6 CG: 0.88+2.5 ADAS-cog (change of score) - After 4 weeks of treatment (p=0.012) IG: -4.2+4.1 CG: 1.1+3.9 - After 12 weeks of treatment (p=0.029) IG: -3.3+5.3 CG: -0.45+6.0 - 12 weeks after discontinuation (p=0.407) IG: -0.26+4.6 CG: -1.4+3.8
Heo et al. (2012)^49^	RCT, parallel, open, controlled South Korea 24 weeks	40 subjects (72.9+9.4 years old, 25% men) Diagnosis of Probable AD by the criteria of NINCDS-ADRDA MMSE≤20	GI-1: 1.5 g de SG-135 a day. GI-2: 3.0 g de SG-135 a day. GI-3: 4.5 g de SG-135 a day CG: only conservative and supportive treatment.	ADAS-cog and MMSE	Subjects from GI-3 presented improvement in the scores ADAS-cog and MMSE in 12 weeks and 24 weeks in comparison with the Baseline. The other groups did not show any difference in any of the periods. ADAS-cog: GI-3 Baseline=41.3+17.0 12 weeks=27.4+ 22.2 (p=0.028) 24 weeks=28.5+23.3 (p=0.028) MMSE: GI-3 Baseline=14.6+6.8 12 weeks=20.8+ 7.2 (p=0.027) 24 weeks=17.0+8.2 (p=0.045)
PHYTOCHEMICALS					
Baumet al. (2008)^50^	RCT, parallel, double-blind, placebo-controlled Hong-Kong 6 months	34 subjects (73.4+8.4 years old, 21% men) Diagnosis of AD by the criteria of NINCDS-ADRDA MMSE=15.4-15.6	IG-1: Turmeric supplement, 4 g a day, tablets or powder. IG-2: Turmeric supplement, 1 g a day, tablets or powder+3 g of placebo powder a day. CG:4 g placebo powder a day.	MMSE	There was no significative difference among the groups in changes of MMSE score after 6 months.
Ringman et al. (2012)^51^	RCT, parallel, single blind, placebo-controlled United States 24 weeks	36 subjects (73.5 years old, 37% men) Diagnosis of AD by the criteria of NINCDS-ADRDA MMSE=17-29	GI-1; 2 g per day of Curcumin C3 Complex*. GI-2: 4 g per day of Curcumin C3 Complex. CG: placebo *Curcumin C3 Complex - formula with 95% Curcuminoids (70-80% Curcumin, 15-25% demetoxicurcumin and 2.5-6.5% Bis-demetoxicurcumin).	ADAS-cog, MMSE.	There was no significant difference between groups in the changes presented in all cognitive parameters after 24 weeks of treatment.
Farokhnia et al. (2014)^52^	RCT, parallel, double-blind Teerã 12 months	68 subjects (77.4+8.0 years old, 57% men) Diagnosis of AD by the criteria of NINCDS-ADRDA MMSE=8-14	IG-1: 10 mg Memantin a day in the first month and 20 mg a day the rest of the period. IG-2: 15 mg per day of dry safflower extract (Crocus Sativus L.) the first month and 30 mg a day the rest of the period.	MMSE and SCIRS	There was no difference among the groups in the changes of scores of MMSE and SCIR after 12 months of treatment.
Gleason et al. (2015)^53^	RCT, parallel, double-blind, placebo-controlled United States 6 months	65 subjects (76.3+7.2 years old, 49% men) Previous diagnosis of AD Diagnostic criteria of AD not described MMSE=22.4-23.5	GI: 100 mg a day of purified soy isoflavone. CG: placebo.	MMSE and Battery of Neuropsychological Tests.	There was no difference among the groups in the MMSE and tests of verbal memory, executive function, executive function and language, visual memory and visuomotor function after 6 months of treatment.
Turner et al. (2015)^54^	RCT, parallel, double-blind, placebo-controlled United States 52 weeks	119 subjects (71.4+7.9 years old, 43% men) Diagnosis of AD by the criteria of NINCDS-ADRDA MMSE=14-26	IG: staggered daily doses of Resveratrol - Weeks 1 to 13: 500 mg - Weeks 14 to 26: 1,000 mg - Weeks 27 to 39: - Weeks 40 to 52: 2,000 mg CG: placebo.	CDR-SOB, ADAS-cog, and MMSE	There was no significative difference among the groups in the scores of CDR-SOB, ADAS-cog, and MMSE (data not provided by the authors).
COCCONUT OIL					
Chan et al. (2017)^55^	RCT, parallel, double-blind, placebo-controlled Malaysia 24 weeks	40 subjects (age between 70 and 79 years old, 15% men) Diagnostic criteria of AD not described MMSE=10-24	IG: coconut oil. - Week 1 and 2: 30 mL per day - Week 3 to 24: 60 mL per day CG: placebo of water with coconut essence. - Weeks 1 and 2: 30 mL per day - Weeks 3 to 24: 60 mL per day	MMSE and CLOX-1	MMSE: In both IG and CG, no significant changes were observed in relation to the baseline after 24 weeks. CLOX-1: - IG: there was no significant difference in relation to the baseline (values not shown). - GC: -0.78571 (p=0.035; 95%CI 1.50824- -0.06319)
PROBIOTIC					
Akbari et al. (2016)^56^	RCT, parallel, double-blind, placebo-controlled Iran 12 weeks	60 subjects (79.8+2.2 years old, 20% men) Diagnosis of AD by the criteria of NINCDS-ADRDA MMSE=8.47-8.67	IG: 200 mL per day of probiotic milk containing Lactobacillus acidophilus, Lactobacillus casei, Bifidobacterium bifidum, and Lactobacillus fermentum (2×109 CFU/g for each). CG: 200 mL per day of milk.	MMSE	Regarding the scores at the baseline, the IG showed a significant improvement in the performance of the MMSE (p<0.001). MMSE - IG: Baseline: 8.67+1.44 12 weeks: 10.57+1.64 MMSE - CG: Baseline: 8.47+1.10 12 weeks: 8.0+1.08
INOSITOL					
Barak et al. (1996)^57^	RCT, cross-over, double-blind, placebo-controlled Israel 8 weeks (4 weeks of cross-over)	12 women (mean age=81.6 years old) Diagnosis of AD by the criteria of DSM-III-R Mild to severe dementia.	IG: 6 g of Inositol per day. CG: placebo (Dextrose).	CAMCOG	There was no difference between groups in changes in CAMCOG. In an analysis by cognitive domains, improvement in orientation and language was observed after 4 weeks of treatment. Orientation (p<0.05) Baseline: 4.09+2.77 4 weeks: 5.36+2.94 Baseline: 4.64+3.26 4 weeks: 4.09+3.33 Language (p<0.05) Baseline: 9.0+5.67 4 weeks: 11.0+6.60 Baseline: 10.64+7.67 4 weeks: 10.55+7.06

ALA: α-lipoic Acid; AD: Alzheimer’s disease; ADAS-cog: Alzheimer's Disease Assessment Scale-Cognitive Subscale; ADAS-cog/11: Alzheimer's Disease Assessment Scale 11-item Cognitive Subscale; CAMCOG: Cognitive Subscale of Cambridge Mental Disorder of the Elderly Examination; CASI: Cognitive Abilities Screening Instrument; CDR-SOB: Clinicial Dementia Rating Scale - Sum of Boxes; CG: control group; CLOX-1: Clock Drawning Test; CoQ: Q Coenzyme; DHA: docosaexaenoic acid; RCT: Randomized Controled Trial; EPA: Eicosapentaenoic Acid; GDS: Global Deterioration Scale; IG: Intervention Group; IWG-1=International Working Group; MEC: Mini Examen Cognitivo; MMSE: Mini Mental State Examination; MRI: magnetic resonance imaging; NIA-AA: National Institute of Aging - Alzheimer Association; NINCDS-ADRDA: National Institute of Neurological and Communicative Disorders and Stroke and the Alzheimer's Disease and Related Disorders Association; NTB: Neuropsychological Test Battery; ONS: oral nutritional supplement; SCIRS: Severe Cognitive Impairment Rating Scale; Set: Isaacs Set Test; SG: Sun Ginseng, WMS-r: Wichsler Memory Scale - revised.

Of the 32 RCT included, one (3%) had a cross-over design,^57^ 26 (87%)^27^ ^,^ ^29^ ^,^ ^31^ ^,^ ^32^ ^,^ ^33^ ^,^ ^34^ ^,^ ^35^ ^,^ ^36^ ^,^ ^37^ ^,^ ^38^ ^,^ ^39^ ^,^ ^40^ ^,,^ ^41^ ^,^ ^42^ ^,^ ^43^ ^,^ ^44^ ^,^ ^45^ ^,^ ^46^ ^,^ ^47^ ^,^ ^50^ ^,^ ^51^ ^,^ ^53^ ^,^ ^54^ ^,^ ^55^ ^,^ ^56^ ^,^ ^57^ were placebo-controlled, five (16%)^26^ ^,^ ^28^ ^,^ ^30^ ^,^ ^48^ ^,^ ^49^ used a conventional control treatment, and one (3%)^52^ compared dietary intervention with pharmacological treatment. Regarding the blinding scheme, 26 (81%) trials were double-blind,^27^ ^,^ ^29^ ^,^ ^31^ ^,^ ^32^ ^,^ ^33^ ^,^ ^34^ ^,^ ^35^ ^,^ ^36^ ^,^ ^37^ ^,^ ^38^ ^,^ ^39^ ^,^ ^40^ ^,^ ^41^ ^,^ ^42^ ^,^ ^44^ ^,^ ^45^ ^,^ ^46^ ^,^ ^47^ ^,^ ^50^ ^,^ ^51^ ^,^ ^52^ ^,^ ^53^ ^,^ ^54^ ^,^ ^55^ ^,^ ^56^ ^,^ ^57^ four (13%) were open^,^ ^28^ ^,^ ^30^ ^,^ ^48^ ^,^ ^49^ and two (6%) were single blind.^26^ ^,^ ^43^

In most trials (59%), the diagnosis of AD was based on the criteria of the National Institute of Neurological and Communicative Disorders and Stroke and the Alzheimer's Disease and Related Disorders Association (NINCDS-ADRDA) and the Diagnostic and Statistical Manual of Mental Disorders (DSM-III and DSM-IV). In one study,^33^ the International Working Group/National Institute of Aging-Alzheimer's Association (IWG/NIA-AA) criteria were used for the diagnosis of prodromal Alzheimer's.

The intervention period in the studies ranged from three weeks to three and a half years. For the assessment of cognitive performance, different instruments were used, being the Mini-Mental State Examination (MMSE), Alzheimer's Disease Assessment Scale-Cognitive Subscale (ADAS-cog), and Clinical Dementia Rating Scale - Sum of Boxes (CDR-SOB) the most frequent tests adopted.

#### Effect of dietary interventions on the cognition of patients with Alzheimer's disease

The effect of different dietary interventions on the cognition of AD patients evaluated by the studies is presented in Table 2, grouped by the type of intervention. Dietary interventions were grouped as oral nutritional formulations, fatty acids (alone or in combination with other nutrients), micronutrients (alone or in combination with other nutrients), ginseng, phytochemicals, coconut oil, probiotics, and inositol. Details of the effects of each intervention are detailed below.

#### Oral nutritional formulations

Eight parallel RCT^26^ ^,^ ^27^ ^,^ ^28^ ^,^ ^29^ ^,^ ^30^ ^,^ ^31^ ^,^ ^32^ ^,^ ^33^ evaluated the effect of intervention with ONF on the cognitive performance of patients with AD. Sample sizes ranged from 37 to 528 subjects, most of whom had mild cognitive decline (MMSE>15 at baseline). Supplements tested included either liquid or semi-solid formulations, offered one to three times a day.

There was heterogeneity among the studies regarding the macro and micronutrient composition of the supplements, trial design, intervention duration (21 days to 24 months), and cognitive outcomes evaluated.

Three RCT tested the same supplement. In these, the use of omega-3, phospholipids, choline, uridine monophosphate, vitamin E, vitamin C, selenium, vitamin B12, vitamin B6, and folic acid enriched formula resulted in a lower decline in the Wechsler Memory Scale - revised immediate recall^29^ and better memory domain performance in the Neuropsychological Test Battery (NTB)^31^ in patients with mild AD and less worsening in the CDR-SOB in patients with prodromal AD.^33^ Among the other studies, one evaluated the same previous formulation in patients with mild to moderate AD,^32^ three^26^ ^,^ ^27^ ^,^ ^30^ evaluated hypercaloric and hyperprotein supplements with different micronutrient compositions and one analyzed a complete dietary formula based on lyophilized foods.^28^ None of them, however, found statistically significant effects on the cognitive outcomes evaluated.

The randomization method used to allocate participants was described in six studies (Table 3), two of which used computer-generated block randomization,^29^ ^,^ ^33^ two had central randomization from codes,^31^ ^,^ ^32^ one used sequential numbers that were kept in sealed envelopes^26^ and a centralized randomization stratified by the initial body mass index.^28^ Of the eight RCT, two were opened^28^ ^,^ ^30^ and five reported blinding of researchers and outcome evaluators.^27^ ^,^ ^29^ ^,^ ^31^ ^,^ ^32^ ^,^ ^33^ The groups studied were comparable at baseline in seven trials.^26^ ^,^ ^27^ ^,^ ^28^ ^,^ ^29^ ^,^ ^30^ ^,^ ^31^ ^,^ ^32^ Regarding the evaluated outcomes, only one study did not present the results of all pre-established outcomes.^26^ Seven of the trials reported follow-up losses,^26^ ^,^ ^28^ ^,^ ^29^ ^,^ ^30^ ^,^ ^31^ ^,^ ^32^ which was greater than 10% in five studies.^26^ ^,^ ^28^ ^,^ ^29^ ^,^ ^32^ ^,^ ^33^ Among those with losses,^26^ ^,^ ^29^ ^,^ ^30^ ^,^ ^31^ ^,^ ^32^ six performed ITT and one^28^ did not report the type of analysis performed.

**Table 3.** Methodological characteristics of included studies.

Study (year)^ref^	Randomization method	Similar groups in the baseline	Blinding of participants	Blinding of outcome assessors	Blinding of researchers	Follow-up losses (%)	IITT	Selection of outcomes.	Other sources of bias
ORAL NUTRITION SUPPLEMENT									
Lauque et al. (2004)^26^	Sequential numbers stored in sealed envelopes	Yes	Yes	No	No	12%	Yes	Yes	Individuals who received an OS prescription during the intervention were not excluded from the CG.
Planas et al. (2004)^27^	NR	Yes	Yes	Yes	Yes	NR	NR	No	
Salas-Salvadó et al. (2005)^28^	Centralized randomization stratified by initial BMI	Yes	No	No	No	21%	NR	No	Acceptance and quantity of dietary formula consumed was not presented.
Scheltens et al. (2010)^29^	Computer-generated block randomization	Yes	Yes	Yes	Yes	12%	Yes	No	
De Sousa et al. (2012)^30^	NR	Yes	No	No	No	5%	Yes	No	
Scheltens et al. (2012)^31^	Central randomization done from codes	Yes	Yes	Yes	Yes	8%	Yes	No	Daily acceptance of the supplement bottle was not reported
Shah et al. (2013)^32^	Central randomization done from codes	Yes	Yes	Yes	Yes	14%	Yes	No	
Soininen et al. (2017)^33^	Computer-generated block randomization	No	Yes	Yes	Yes	21%	Yes	No	
OMEGA-3 FATTY ACIDS ISOLATED OR IN ASSOCIATION WITH OTHER NUTRIENTS									
Yehuda et al. (1996)^34^	NR	NR	Yes	Yes	NC	0%	Yes	No	
Freud-Levi et al. (2006)^35^	NR	Yes	Yes	NC	NC	15%	No	Yes	
Quinn et al. (2010)^36^	Centralized block randomization using interactive voice response system	No	Yes	Yes	Yes	27%	Yes	No	
Shinto et al. (2014)^37^	Computer-generated randomization scheme stratified by smoking status (current smoker versus nonsmoker)	No	Yes	Yes	Yes	13%	NR	No	
MICRONUTRIENTS ISOLATED OR IN ASSOCIATION									
Sano et al. (1997)^38^	Permuted-block randomization	Yes	Yes	NC	NC	7%	Yes	No	
Sun et al. (2007)^39^	Computer generated random number list	Yes	Yes	Yes	Yes	29%	Yes	No	
Kessler et al. (2008)^40^	NR	Yes	Yes	NC	NC	16%	NR	No	
Aisen et al. (2008)^41^	Permuted-block randomization	Yes	Yes	NC	Yes	16%	Yes	No	
Lloret et al. (2009)^42^	Randomized list of numbers	Yes	Yes	NC	NC	56%	No	No	
Remington et al. (2009)^43^	NR	NR	Yes	NC	NC	58%	Yes	No	
Galasko et al.(2012)^44^	Permuted-block randomization	Yes	Yes	NC	Yes	20%	No	No	Patients were allowed to continue using antioxidant supplements for daily use, as long as within the following limits: <100 IU/day of α-tocopherol; <200 mg/day of C vitamin; 60 mg/day of CoQ; <100 IU/day of ALA
Dyskenet al. (2014)^45^	Central permuted-block randomization	Yes	Yes	NC	Yes	42%	Yes	No	Dose adjustments were allowed based on the participants' tolerance
Nolan et al. (2015)^46^	Computer generated block randomization	No	Yes	NC	NC	15%	NR	Yes	
Remington et al. (2015)^47^	Randomization done from codes	No	Yes	Yes	Yes	78%	Yes	Yes	
GINSENG									
Lee et al. (2008)^48^	NR	Yes	No	No	No	15%	Yes	No	
Heo et al. (2012)^49^	NR	Yes	No	No	No	NR	NR	No	
PHYTOCHEMICALS									
Baum et al. (2008)^50^	NR	Yes	Yes	NC	NC	21%	NR	No	
Ringman et al. (2012)^51^	Block randomization	Yes	Yes	Yes	Yes	17%	No	No	
Farokhnia et al. (2014)^52^	Randomization done from codes	Yes	Yes	Yes	Yes	12%	Yes	No	
Gleason et al. (2015)^53^	NR	Yes	Yes	NC	NC	9%	Yes	No	
Turner et al. (2015)^54^	Permuted-block randomization	No	Yes	NC	Yes	13%	No	Yes	
COCONUT OIL									
Chan et al. (2017)^55^	Block randomization	Yes	Yes	NC	Yes	45% (most in IG)	No	Yes	
PROBIOTIC									
Akbari et al. (2016)^56^	Computer generated random number list	Yes	Yes	NC	Yes	10%	Yes	No	
INOSITOL									
Barak et al. (1996)^57^	NR	NR	Yes	NC	NC	8%	No	No	

ALA: α-lipoic acid; ITT: intention-to-treat analysis; CG: control group; IG: intervention group; BMI: body mass index; NC: not clear; NR: not reported; OS: oral supplementation.

#### Fatty acids

Four parallel RCT^34^ ^,^ ^35^ ^,^ ^36^ ^,^ ^37^ tested the effect of fatty acid supplementation alone or associated with other nutrients on cognitive outcomes in individuals with AD. The duration of interventions varied between 4 weeks and 18 months and the trials were heterogeneous in relation to the sample size (39‒582 participants per trial), dose used, presence of vitamin E and α-lipoic acid (ALA), composition and fatty acid content (isolated docosahexaenoic acid (DHA), DHA+eicosapentaenoic acid (EPA) and mixture of α-linolenic acid with linoleic acid).

In all four studies, fatty acid supplementation resulted in significant improvement in part of the cognitive parameters analyzed. Twelve-month supplementation with 5,800 mg per day of omega-3 associated with vitamin E in patients with mild AD was effective in promoting a lower rate of cognitive decline on MMSE, but only in the first six months of treatment.^35^ Smaller decline on MMSE was also observed with the use of omega-3 associated with ALA in mild AD (3,000 mg of omega-3+600 mg of ALA per day)^37^ and the use of seaweed-derived DHA (2,000 mg per day, 45‒55% of DHA) has shown to be beneficial for patients with negative Apoliprotein E (APOE) ε4 allele, being able to reduce the cognitive decline assessed by ADAS-cog.^36^

In patients with advanced AD (baseline MMSE=7.8+3.8), the use of 2 mL per day of a formulation containing a mixture of α-linolenic and linoleic in a ratio of 1:4.5 combined with α-tocopherol resulted in more reports by caregivers of improvement in the patients’ general condition.^34^

Regarding methodological quality, one of the trials used a block randomization method based on an interactive voice response system with stratification by center,^36^ one had computer-generated randomization with stratification by smoking,^37^ and the others did not describe the randomization method used to allocate participants.^34^ ^,^ ^35^ The groups studied were comparable at baseline in only one^35^ of the four RCT, and one of the remaining RCT this data was not reported.^34^ Three trials^35^ ^,^ ^36^ ^,^ ^37^ showed follow-up losses, all greater than 10%. Of these, 36 performed the ITT and one did not report the type of analysis performed.^34^ Regarding the evaluated outcomes, only one study^37^ did not present the results of all pre-established outcomes.

#### Micronutrients

Ten parallel RCT evaluated micronutrient supplementation alone or in combination compared to placebo^39^ ^,^ ^40^ ^,^ ^41^ ^,^ ^42^ ^,^ ^43^ ^,^ ^44^ ^,^ ^46^ ^,^ ^47^ or pharmacological treatment.^38^ ^,^ ^45^ There was heterogeneity in relation to blinding (nine double-blind and one-blind), sample size (12-613 participants per trial), duration of intervention (16 weeks to 4 years), assessed cognitive outcomes and nutrients used (α-tocopherol alone,^38^ ^,^ ^42^ ^,^ ^45^ B^41^ vitamins association, carotenoid association,^46^ copper,^58^ coenzyme Q,^44^ multivitamin and mineral supplement,^39^ vitamins associated with ALA^44^ and nutraceutical formulation containing vitamins, minerals, and peptides).^43^ ^,^ ^47^

In patients with mild AD (MMSE=22.2+5.1), the use of a nutraceutical formula containing folic acid, vitamin B12, α-tocopherol, S-adenosyl methionine, N-acetyl cysteine, ​​and acetyl-L-carnitine resulted in significant improvement in the Clock Drawing Test (CLOX-1) and the Age- and Education-adjusted Dementia Rating Scale (DRS-AEMSS) scores compared to baseline.^47^ Such result differs from the pilot RCT performed with the same formulation, where no significant differences were found between groups in DRS-2 and CLOX-1.^43^

Copper orotate supplementation was also evaluated in a group of patients with mild AD (MMSE=20‒25). Both the intervention and control groups showed an increase in the ADAS-cog score and a reduction in MMSE performance in relation to the baseline. These results were statistically significant. In the comparison between groups, the increase in ADAS-cog was smaller in the supplemented group, indicating lower cognitive decline. There was no difference between the groups regarding MMSE performance.^58^

In patients with mild to severe AD, supplementation with vitamin E alone (800 IU/day) proved to be ineffective and, for some participants, harmful. Comparing intervention and control groups, supplement use did not alter cognitive performance in the MMSE, CLOX-1, and Blessed Dementia Scale. However, patients that at the end of the intervention did not obtain a reduction on serum oxidized glutathione levels, considered “unresponsive” to treatment with vitamin E, had a significant decline in MMSE when compared to “responsive” ones.^42^

Similar results in MMSE performance were also found with vitamin E supplementation (800 IU/day) associated with vitamin C (500 mg/day) and ALA (900 mg/day).^44^ Compared to the control, the supplemented group showed a significant reduction in MMSE performance. Paradoxically, in the same group, there was a decline in levels of F2-isoprostane relative to baseline, a biomarker of oxidative damage.

Of the five other studies, two compared α-tocopherol supplementation with pharmacological treatment (Selegiline^38^ and Memantine^45^), one analyzed the effect of methylcobalamin supplementation in combination with multivitamin,^39^ one used a combination of folic acid, vitamin B12 and pyridoxine^41^ and one a carotenoid supplement containing mezo-zeaxanthin, lutein, and zeaxanthin.^46^ In none of these trials, however, the results found were significant for the cognitive parameters evaluated.

The randomization method used to allocate participants was described in eight of the ten studies. Of these, five^16^ ^,^ ^38^ ^,^ ^41^ ^,^ ^44^ used permuted block randomization, two^39^ ^,^ ^42^ were randomized using a list of random numbers, and one^47^ used code randomization.

The studied groups were comparable at baseline in seven^38^ ^,^ ^39^ ^,^ ^40^ ^,^ ^41^ ^,^ ^42^ ^,^ ^44^ ^,^ ^45^ RCT, and in two^43^ ^,^ ^37^of the remainder these data were not reported. All trials showed follow-up losses greater than 10% for the most part of the studies.^39^ ^,^ ^40^ ^,^ ^41^ ^,^ ^42^ ^,^ ^43^ ^,^ ^44^ ^,^ ^45^ ^,^ ^46^ ^,^ ^47^ Five studies^38^ ^,^ ^41^ ^,^ ^43^ ^,^ ^45^ ^,^ ^47^ performed the ITT and two did not report the type of analysis performed.^58^ ^,^ ^46^ Regarding the evaluated outcomes, two studies^46^ ^,^ ^47^ did not present the results of all pre-established outcomes.

#### Ginseng

Two open parallel trials evaluated ginseng supplementation in individuals with AD.^48^ ^,^ ^49^ The duration of interventions varied between 12 and 24 weeks and the studies were heterogeneous in terms of sample size (58‒97 subjects), doses, and types of ginseng used.

In both trials, ginseng supplementation resulted in significant improvement in the cognitive outcomes evaluated. Patients with moderate to severe AD (MMSE<20 and CDR score>1) treated with 4.5 g/d of Sun Ginseng (SG-135) showed significant improvement in the ADAS-cog and MMSE after 12 and 24 weeks of supplementation.^49^ Similar results were found with the use of 4.5 and 9.0 g/d of Korean white ginseng in a sample of patients with mild to moderate AD. Compared to control, both doses resulted in improvement in the MMSE and ADAS-cog scores after 12 weeks of supplementation and such effect was eliminated 12 weeks after discontinuation of treatment.^49^

The groups studied were comparable at baseline in both RCT. A follow-up loss of 15% was reported by one of the trials, which performed an ITT analysis.^48^ In the other trial, information about follow-up losses and data analysis method were not reported.^49^ In both trials, the results of all predetermined outcomes were presented.

#### Phytochemicals

Five parallel double-blind RCT^50^ ^,^ ^51^ ^,^ ^52^ ^,^ ^53^ ^,^ ^54^ evaluated the effect of supplementation of different phytochemicals extracted from foods and condiments compared with placebo^50^ ^,^ ^51^ ^,^ ^53^ ^,^ ^54^ or pharmacological treatment.^52^ Of the five trials, two evaluated the effect of turmeric at different doses and concentrations of curcuminoids,^50^ ^,^ ^51^ one used dried turmeric extract,^52^ one purified soy isoflavone,^53^ and another, steady doses of Resveratrol.^54^ In none of the trials, however, the results found were significant for the cognitive parameters evaluated.

Heterogeneity among studies was observed in relation to sample size (34‒119 subjects per trial), degree of cognitive decline (mild to severe), duration of the intervention (6 to 13 months), and cognitive outcomes (ADAS-cog, CDR-SOB, MMSE, and Severe Cognitive Impairment Rating Scale - SCIRS).

The randomization method used to allocate participants was described in three of the five studies. Of these, two^51^ ^,^ ^54^ used block randomization and one^52^ had randomization carried out from codes that were kept in opaque, sealed, and sequentially numbered envelopes.

The groups studied were comparable at baseline in four RCT.^50^ ^,^ ^51^ ^,^ ^52^ ^,^ ^53^ All trials had follow-up losses greater than 10% in most studies.^50^ ^,^ ^51^ ^,^ ^52^ ^,^ ^54^ Of the five trials, two^52^ ^,^ ^53^ performed ITT and one did not report the type of analysis performed.^50^ As for the outcomes evaluated, four of the five studies^50^ ^,^ ^51^ ^,^ ^52^ ^,^ ^53^ presented the results of all pre-established outcomes.

#### Coconut oil

A parallel, double-blind, placebo-controlled RCT^55^ evaluated the effect of coconut oil on the cognitive performance of subjects with mild to moderate AD. Using the block randomization method, 58 subjects were randomized to receive coconut oil or placebo. The characteristics of the groups were similar at baseline. After 6 months of intervention, no change was observed in the cognitive performance assessed by MMSE and CLOX-1.

Regarding the methodological quality, the study showed a 45% loss of follow-up, which was higher in the intervention group due to side effects such as diarrhea and abdominal discomfort. Data analysis was performed per protocol and the results of all pre-established outcomes were not presented.

#### Probiotics

A parallel, double-blind, placebo-controlled RCT^56^ assessed the effect of probiotic supplementation onIN 60 patients with advanced AD. Using a computer program to generate a random list, participants were randomly allocated to receive milk with probiotics (L. Acidophilus, L. Casei. B. Bifidum, and L. Fermentum) or placebo. After 12 weeks of intervention, the probiotic supplemented group had a significant improvement in MMSE performance.

Regarding methodological quality, the groups were different at baseline in relation to some metabolic parameters (triglyceride levels, high density lipoprotein -HDL, and very low density lipoprotein - VLDL), but had similar cognitive characteristics. The follow-up loss was 10% and data were analyzed by ITT. The blinding of participants and researchers was maintained until the analysis conclusion and all the pre-established outcomes were reported.

#### Inositol

The effect of inositol supplementation was evaluated on a double-blind, placebo-controlled crossover RCT.^57^ Twelve women with mild to severe AD were randomly allocated to receive inositol or dextrose (placebo). After 4 weeks of treatment, supplementation resulted in a significant improvement in the orientation and language domains assessed by the Cognitive Subscale of Cambridge Mental Disorder of the Elderly Examination (CAMCOG).

Regarding the methodological quality, the randomization method used was not reported in the study, there was a loss of 8% follow-up and data analysis was performed per protocol.

## DISCUSSION

This systematic review aimed to evaluate the effect of different dietary interventions on the management of cognitive decline in AD patients.

Our study indicates the effects of improving or delaying cognitive decline with the use of specialized nutritional formulas, fatty acid supplements, ginseng, inositol probiotics. However, it is noteworthy that such results were mostly obtained in patients with mild AD, limited to only one part of the cognitive outcomes evaluated and resulted from the use of associated and not isolated nutrients in most trials, which suggests that the observed effects may (or may not) be due to the association of rather than a single target nutrient.

Regarding specialized oral nutritional formulations, in the prodromal phase and in the early stages of the disease, Fortasyn Connect (Souvenaid®), an oral supplement that includes a combination of EPA, DHA, phospholipids, uridine monophosphate, choline, selenium, and vitamins B6, B12, B9, C, and E, showed good results in the patients’ cognitive performance.

Suggested mechanisms for the effects of Fortasyn Connect include increased bioavailability of precursors and co-factors required for neuronal formation, maintenance and function, increased acetylcholine levels and cholinergic receptors with consequent stimulation of synaptogenesis and reduction of Aβ production and neurotoxicity.^58^ However, despite the promising therapeutic effect of prodromal AD, the effects of supplementation with Fortsasyn Connect are still divergent in patients with mild to moderate disease, and further studies are needed to elucidate differences in the outcomes and to confirm the existence of therapeutic benefits on cognition.

The neuroprotective action of omega-3 fatty acids, especially DHA, has been demonstrated in several *in vitro* experiments and in animal models of AD, reinforcing the idea that supplementation of these nutrients could help reduce neuroinflammation and cognitive decline.^59^ In mouse models of AD, DHA treatment resulted in reduced brain levels of Aβ, particularly β-Amyloid 42 (Aβ-42), the main component of amyloid plaques that contributes to irreversible neuronal death and rapid disease progression. Other mechanisms involved in the action of DHA include anti-inflammatory, antioxidant, and anti-apoptotic effects.^59^

Neuroinflammation, chronic activation of glial cells and increased production of reactive oxygen species are involved in the pathogenesis and progression of AD. In *in vitro* studies and in patients with AD, DHA administration was able to induce microglial phagocytosis of Aβ-42, decrease IL-1beta, IL-6 production and the activation of proinflammatory transcription of the nuclear factor Kappa B (NFκB).^59^ In animal models of AD, treatment with DHA has also been shown to increase levels of antioxidant enzymes catalase and glutathione peroxidase, as well as to reduce oxidative damage to the cerebral cortex and hippocampal cells.^59^

Oxidative stress-induced by Aβ deposition is primarily responsible for TP hyperphosphorylation, which is associated with neuronal damage and induction of an apoptotic cascade.^59^ In addition to the antioxidant effects already mentioned, DHA performance includes the regulation of the apoptotic cascade induced by Aβ at the level of lipid peroxides, conferring neuroprotection to neuronal cells. However, the effects of DHA on cognitive function and AD progression are absent in individuals who possess the APOE ε4 allele, which is associated with lower DHA uptake in the brain.^59^

In the three studies that evaluated the use of omega-3 in AD,^35^ ^,^ ^36^ ^,^ ^37^ supplementation was effective in delaying cognitive decline in individuals with AD, but there was a significant variation between studies in relation to the doses, proportion of EPA and DHA and association of antioxidants (only one study used DHA alone). Adverse effects were also reported in the studies, including changes in the International Normalized Ratio (INR) in subjects taking warfarin^36^ and with diarrhea.^35^ ^,^ ^37^

Improvement in several cognitive domains was also observed in a study of patients with advanced stages of the disease who were supplemented with a mixture of α-Linolenic fatty acids and Linoleic acid.^34^ However, some methodological flaws of the study should be considered, including the use of an invalidated instrument for cognitive assessment and the absence of statistical treatment adjusting for confounding factors. Thus, other studies with greater methodological rigor are necessary to confirm the efficacy in the cognitive improvement of patients with AD.

Among micronutrient supplements, only supplementation with formula containing folic acid, vitamin B12, α-tocopherol, S-adenosyl methionine, N-acetyl cysteine, ​​and acetyl-L-carnitine showed positive results in the cognitive performance of AD patients.^47^ In *in vitro* experiments and in animal models of AD, the administration of such components has been associated with reduced oxidative stress and decreased Aβ production and TP phosphorylation.^43^ However, such results should be interpreted with caution due to limitations and methodological flaws of the study.

Ginsenoside administration in AD animal models has been shown to be associated with a neuroprotective effect and better memory performance.^60^ In the two studies in this review that evaluated Ginseng supplementation,^48^ ^,^ ^49^ the treated groups demonstrated better cognitive performance in ADAS-cog and MMSE. However, limitations in the methodological quality of the trials do not allow us to draw a conclusion about the benefits found. Therefore, further studies with better methodological quality are necessary to evaluate the use of Ginseng supplementation in individuals with AD.

In individuals with advanced AD, supplementation for three months of a probiotic milk containing Lactobacillus acidophilus, Lactobacillus casei, Bifidobacteriumbifidum, and Lactobacillus fermentum resulted in improved performance in MMSE, but the mechanisms for this finding still need clarification.^56^ Thus, further studies are needed to confirm this therapeutic potential and to elucidate the mechanism by which probiotics interfere with the neurodegenerative process of AD.

The use of inositol resulted in improved performance of AD patients in orientation and language cognitive domains.^57^ However, this effect should be interpreted with caution once it was only a small trial and the sample was heterogeneous in relation to educational level, time of diagnosis and phase of dementia, factors that are known to influence cognitive performance. Therefore, further studies are needed to confirm a possible benefit with inositol supplementation.

Other interventions included the use of B-complex vitamins, Copper Orate, α-tocopherol, carotenoids, turmeric, soy isoflavones, resveratrol, and coconut oil, but there was no evidence of benefit in cognition in the studies.

The limitations of this review should be considered and include the limitation of articles in English and the impossibility of performing a meta-analysis due to methodological differences and the limited extent of available literature.

The strengths of this paper should also be highlighted and include the search in three databases, reading of titles and abstracts by two researchers and unlimited search for the date of publication of the articles.

The present systematic review points out that the effect of most dietary interventions on cognition in AD patients is inconclusive due to limited scientific evidence due to the poor methodological quality of the primary studies and the reduced number of studies. However, several nutrients associated and isolated DHA derived from algae show potential to improve cognitive function in AD, especially in its early stages. Thus, in a challenging scenario with a significant increase in the number of diagnoses of AD, better quality studies are urgently needed to confirm the therapeutic potential of the diet so that a dietary recommendation in AD that contributes to the quality of life of patients and relatives can be established.
